# Very Late Recurrence of Clear Cell Renal Cell Carcinoma With Pancreatic and Hepatic Metastases Showing Durable Response to Dual Checkpoint Blockade

**DOI:** 10.1002/ccr3.71852

**Published:** 2026-01-14

**Authors:** Fumihiro Ito, Koki Kobayashi, Gaku Hayashi, Shunsuke Kamijo, Takashi Fujita

**Affiliations:** ^1^ Department of Urology Gifu Prefectural Tajimi Hospital Tajimi Japan

**Keywords:** clear cell renal cell carcinoma, ipilimumab, liver neoplasms/secondary, nivolumab, pancreatic neoplasms/secondary

## Abstract

Very late recurrence of clear cell renal cell carcinoma with pancreatic involvement is uncommon, and treatment and toxicity management are not well defined. A patient developed liver and pancreatic lesions approximately 18 years after nephrectomy for clear cell renal cell carcinoma. Liver biopsy with immunohistochemistry confirmed metastatic renal origin; the pancreatic mass was diagnosed radiologically. Nivolumab plus ipilimumab (four induction cycles) followed by nivolumab maintenance induced a partial response. During maintenance, the patient experienced an immune‐related endocrine adverse event with secondary adrenal insufficiency and central hypothyroidism, managed with hormone replacement without treatment interruption. Performance status remained excellent, and tumor response has been durable for more than 30 months. This case highlights that endocrine immune‐related adverse events can be safely managed with appropriate hormone replacement, allowing uninterrupted immunotherapy and preserving long‐term benefit. Dual immune checkpoint blockade can achieve durable disease control in very late recurrent clear cell renal cell carcinoma with pancreatic and hepatic metastases, and endocrine toxicity may be safely managed to maintain benefit.

## Key Clinical Message

Dual immune checkpoint blockade can achieve long‐term disease control in very late recurrent clear cell renal cell carcinoma involving pancreas and liver, when immune‐related endocrine toxicity is promptly recognized and managed with hormone replacement.

## 1 Introduction

Very late recurrence (≥ 10 years) of clear cell renal cell carcinoma (ccRCC) is exceptionally rare, representing less than 1% of cases in long‐term follow‐up cohorts [1]. Pancreatic metastasis from RCC is a distinctive but infrequent manifestation that often follows an indolent course, whereas hepatic involvement is typically associated with poor prognosis [2]. The coexistence of pancreatic and hepatic metastases after nearly two decades of disease‐free survival poses both diagnostic and therapeutic challenges, and durable responses in this setting have scarcely been documented.

Combination immune checkpoint blockade with nivolumab plus ipilimumab has established itself as a standard first‐line regimen for intermediate‐ or poor‐risk advanced ccRCC, providing superior survival and long‐term durability compared with sunitinib [3, 4, 5]. In parallel, checkpoint inhibitor plus tyrosine‐kinase inhibitor combinations, such as pembrolizumab plus axitinib, have also demonstrated significant efficacy [6]. However, the role of dual checkpoint blockade in very late recurrent ccRCC with multi‐organ involvement remains largely unexplored.

We describe a rare case of very late recurrent ccRCC—occurring 18 years after nephrectomy—with simultaneous pancreatic and hepatic metastases. Treatment with nivolumab plus ipilimumab induced a durable partial response lasting over 30 months, during which immune‐related endocrine adverse events were successfully managed without treatment interruption.

## 2 Case History/Examination

A man with a history of left nephrectomy for pT1b clear cell RCC presented with systemic symptoms and was found to have multiple liver lesions (largest 76 mm), a pancreatic body mass (largest 83 mm), and enlarged hepatoduodenal ligament nodes on cross‐sectional imaging. Baseline performance status was excellent (ECOG 0/Karnofsky 100%), and the International Metastatic Renal Cell Carcinoma Database Consortium (IMDC) risk category was intermediate (anemia and thrombocytosis) [7].

**FIGURE 1:**
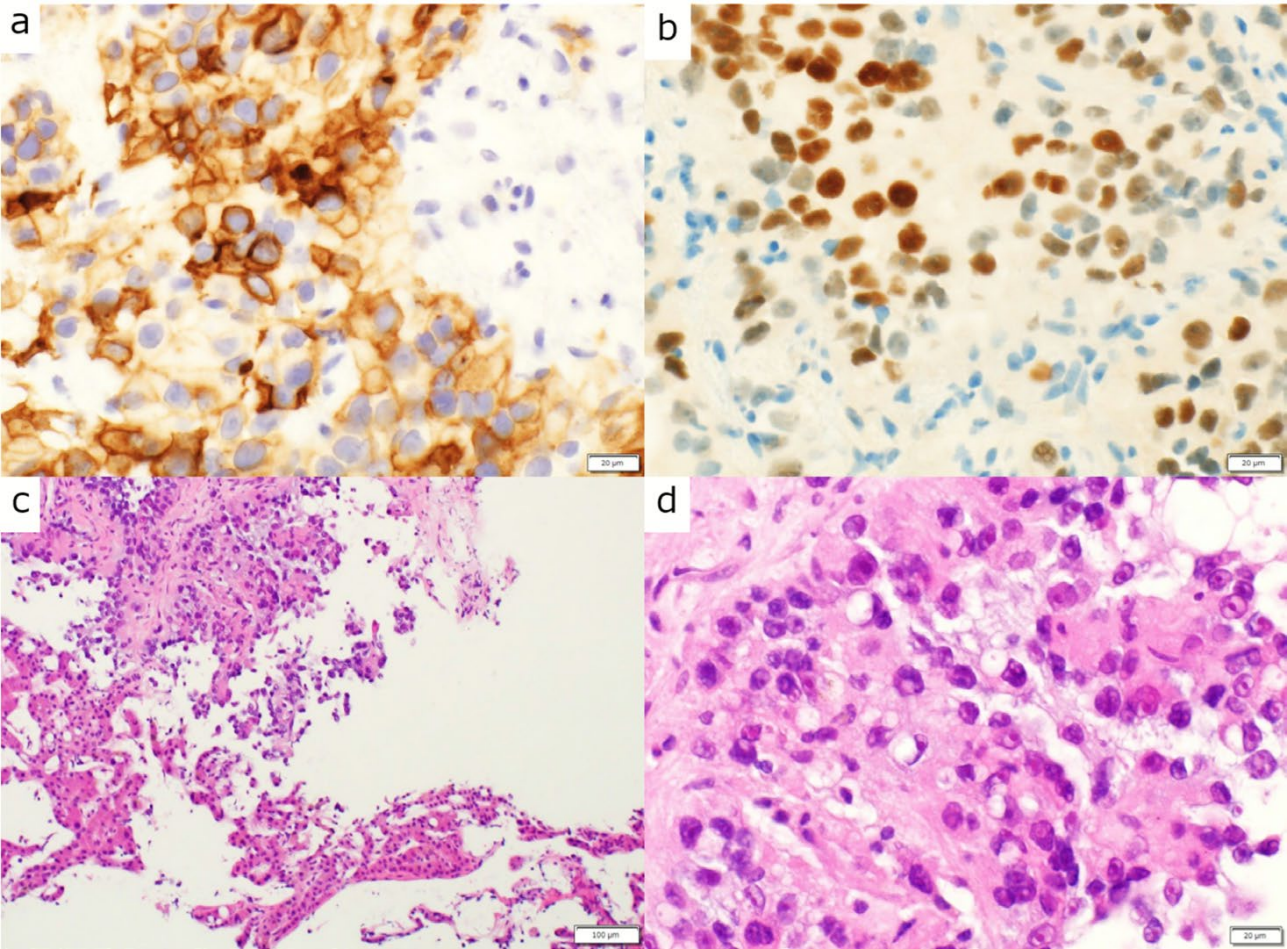
Histopathologic findings of the liver biopsy confirming metastatic clear cell RCC. (a) CD10 positive, (b) PAX8 positive, (c) hematoxylin–eosin (H&E) staining at ×100 magnification, and (d) H&E staining at ×400 magnification. Scale bars: 20 μm in panels a, b, d; 100 μm in panel c.

Liver biopsy revealed metastatic RCC (Figure 1), while the pancreatic lesion was diagnosed radiologically.

**FIGURE 2:**
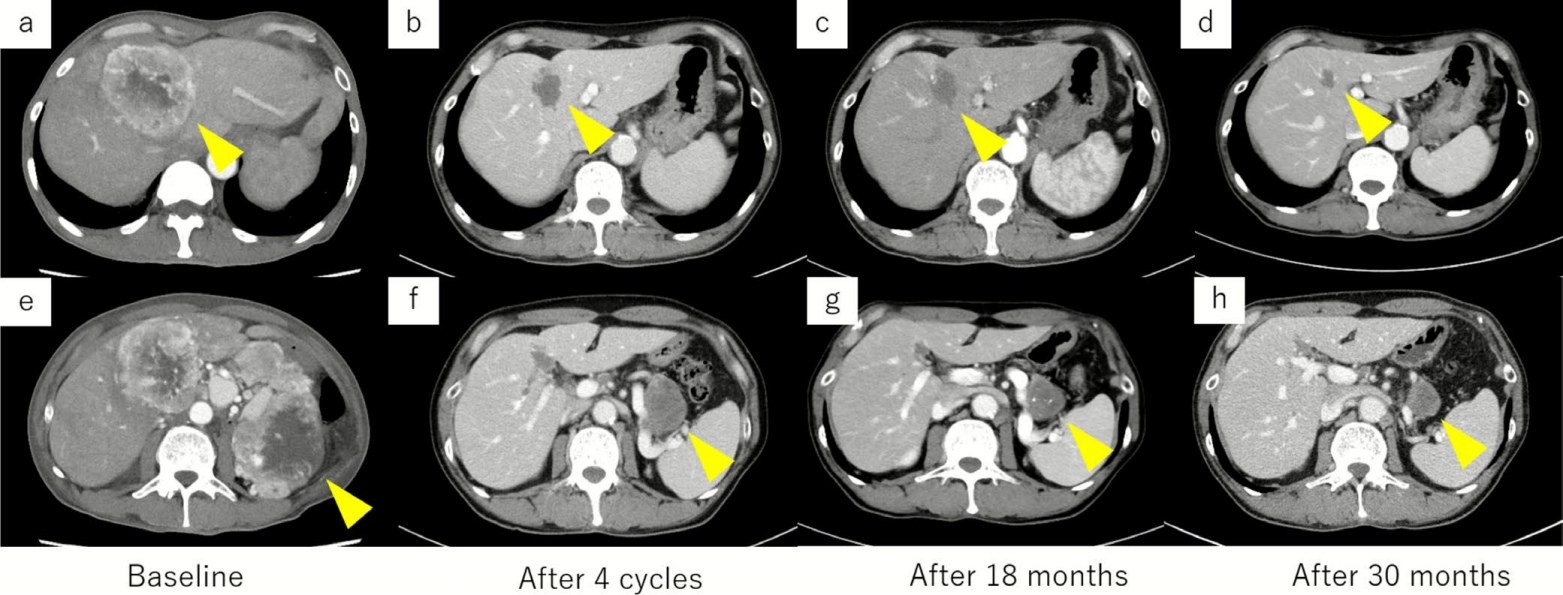
Radiologic timeline demonstrating partial response and durable control on nivolumab + ipilimumab. (a–d) Liver metastases: Baseline, partial response after 4 cycles, 18 months, and 30 months showing sustained regression. (e–h) Pancreatic lesion: Corresponding time points showing concordant shrinkage and long‐term stability.

The patient received nivolumab plus ipilimumab induction (four 3‐weekly cycles) followed by nivolumab maintenance at standard dose and interval. During treatment, he developed endocrine immune‐related adverse events presenting with fatigue and biochemical evidence of secondary adrenal insufficiency and central hypothyroidism. Hormone replacement with hydrocortisone and levothyroxine effectively controlled symptoms, allowing continued therapy. Performance status remains excellent, and partial response has been maintained for over 30 months (Figure 2).

## 3 Methods (Differential Diagnosis, Investigations and Treatment)

Differential diagnoses included primary pancreatic carcinoma, neuroendocrine tumor, and metastatic renal cell carcinoma.

Histopathologic examination of the liver biopsy confirmed metastatic clear cell RCC, showing PAX8 and CD10 positivity (Figure 2).

Based on these findings, combination immunotherapy with nivolumab and ipilimumab was initiated, followed by nivolumab maintenance according to standard dosing.

## 4 Conclusions and Results (Outcome and Follow‐Up)

**FIGURE 3:**
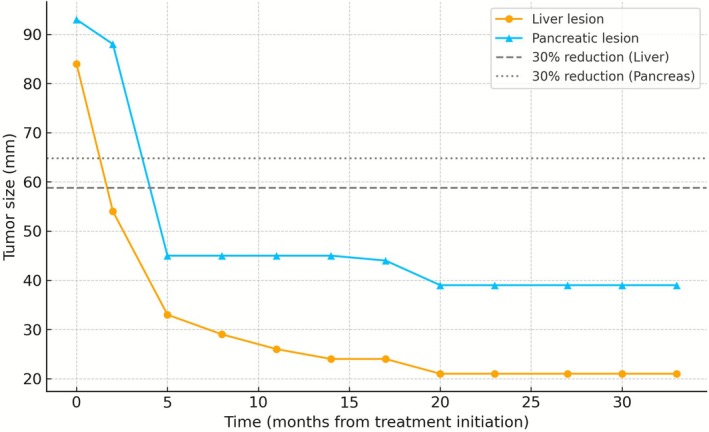
Quantitative change in target lesion size over time according to RECIST 1.1. The dotted lines indicate the 30% reduction threshold for partial response for each organ (53 mm for liver, 58 mm for pancreas). Both lesions achieved partial response within 4 cycles and remained stable for over 30 months.

A partial response was achieved after induction and has been sustained for over 30 months (Figure 2). Serial CT imaging demonstrated concordant shrinkage of both liver and pancreatic lesions (Figure 3). The patient developed immune‐related endocrine dysfunction—secondary adrenal insufficiency and central hypothyroidism—which was successfully managed with hormone replacement, allowing uninterrupted immunotherapy.

The patient's performance status has remained excellent, and no disease progression has been observed to date.

## 5 Discussion

This case links three clinically relevant themes: (i) very late recurrence of clear cell RCC after a prolonged disease‐free interval, (ii) pancreatic organotropism occurring alongside multiple hepatic metastases, and (iii) sustained, multi‐year disease control on ipilimumab plus nivolumab (IPI + NIVO) despite the traditionally adverse prognostic signal of liver involvement. While the coexistence of a pancreatic target lesion and multiple liver metastases would usually suggest a challenging clinical scenario, an early partial response (PR) was followed by durable control without treatment interruption, illustrating that deep early responses on dual checkpoint inhibition can be long‐lived [3, 4, 5].

The durability of early partial responses with dual checkpoint blockade is further supported by extended analyses from CheckMate 214, which consistently show long‐term disease control and survival plateaus on ipilimumab plus nivolumab across multiple follow‐ups [6, 8, 9].

### 5.1 Very Late Recurrence and Surveillance Implications

Metastatic relapse more than a decade after nephrectomy, as observed here 18 years post‐surgery, is rare but increasingly recognized [1]. Such very late recurrence raises important questions about long‐term surveillance. Universal CT follow‐up beyond 10 years is unlikely to be cost‐effective or feasible; however, clinicians should maintain vigilance for non‐specific systemic symptoms and pursue prompt investigation when they occur. A risk‐adapted, individualized surveillance strategy that incorporates baseline pathology, patient comorbidities, and preferences appears most reasonable in this setting.

### 5.2 Pancreatic Organotropism and Synchronous Liver Metastases

Pancreatic metastases from RCC (PM‐RCC) are uncommon, often hypervascular, and radiologically mimic neuroendocrine tumors. Reports suggest that PM‐RCC may follow a more indolent course than metastases to other organs, consistent with the “seed‐and‐soil” hypothesis and tumor dormancy [2]. Conversely, liver metastases are traditionally associated with inferior survival [2]. The coexistence of these opposing metastatic behaviors in one patient underscores the heterogeneity of RCC biology.

Although histologic confirmation of the pancreatic lesion was not obtained, the unified disease origin was well supported by the liver biopsy findings (PAX8 and CD10 positivity), the classic hypervascular pancreatic imaging pattern, and the concordant shrinkage of both lesions under dual checkpoint blockade. This limitation is acknowledged.

Notably, our observation of durable control despite liver involvement contrasts with historical data associating hepatic metastases with inferior outcomes in RCC [2], underscoring that immunotherapy can occasionally overcome site‐specific adverse biology. Notably, the liver disease was multifocal with a dominant 7.6‐cm lesion, yet a durable partial response was achieved, underscoring that dual ICI can occasionally overcome the traditionally adverse prognostic signal of hepatic involvement even in bulky, multi‐lesional settings.

### 5.3 Management of Immune‐Related Endocrine Toxicity

Dual checkpoint blockade provides durable benefit but also increases the risk of immune‐related adverse events (irAEs), particularly endocrine dysfunction when CTLA‐4 inhibition is included [10, 11]. In this case, secondary adrenal insufficiency and central hypothyroidism developed but were effectively controlled with glucocorticoid and levothyroxine replacement. Importantly, systemic therapy was not discontinued, and the patient sustained a partial response for more than 30 months. This highlights the principle that timely recognition and appropriate hormone replacement can allow continuation of immunotherapy, avoiding premature cessation and preserving long‐term benefit [10, 11].

### 5.4 Broader Implications

Compared with the historical VEGF‐targeted standards of care, such as bevacizumab plus interferon‐α and sunitinib [12, 13], dual immune checkpoint blockade offers a distinct therapeutic paradigm characterized by the possibility of deep, durable responses and treatment‐free plateaus in a subset of patients.

This case illustrates that dual ICI can secure durable disease control even in patients with pancreatic and hepatic metastases, provided an early deep response is achieved and toxicities are proactively managed. It also emphasizes the need for risk‐adapted long‐term surveillance after nephrectomy, careful differential diagnosis of hypervascular pancreatic lesions, and structured management of endocrine irAEs. Together, these insights add to the growing recognition that metastatic RCC remains clinically heterogeneous and that durable responses to immunotherapy can emerge even in high‐burden scenarios once effective immune reactivation is achieved. Although modern ICI–TKI combinations provide high initial response rates [14, 15], our case illustrates that durable benefit from dual ICI can extend beyond conventional IMDC risk boundaries, even in patients with traditionally adverse sites such as the liver. Few reports have described concurrent pancreatic and hepatic metastases achieving such prolonged control, underscoring the potential of dual checkpoint blockade in atypical metastatic patterns.

In conclusion, this case highlights that metastatic recurrence of clear cell RCC can occur even two decades after nephrectomy and that dual immune checkpoint blockade can achieve durable disease control despite synchronous pancreatic and hepatic metastases. Vigilant long‐term follow‐up and proactive toxicity management are crucial to optimize outcomes in such atypical late recurrences.

## Author Contributions

**Fumihiro Ito:** conceptualization, data curation, formal analysis, funding acquisition, investigation, methodology, project administration, resources, software, validation, visualization, writing – original draft. **Koki Kobayashi:** investigation. **Gaku Hayashi:** investigation. **Shunsuke Kamijo:** investigation. **Takashi Fujita:** supervision, writing – review and editing.

## Funding

The authors have nothing to report.

## Ethics Statement

Ethical approval was not required for this single‐patient case report in accordance with institutional policies.

## Consent

Written informed consent for publication of this case and accompanying images was obtained from the patient.

## Conflicts of Interest

The authors declare no conflicts of interest.

## Data Availability

Data sharing is not applicable to this article as no new datasets were generated or analyzed during the current study.
